# Absolute quantification by mouthwash testing screens severe periodontitis and tracks species-specific microbial response to periodontal therapy

**DOI:** 10.3389/fcimb.2026.1917870

**Published:** 2026-09-03

**Authors:** Je-Hyun Eom, Mu-Yeol Cho, Ji-Won Kim, Yunwoo Kim, Jeehyun No, Jiyoung Hwang, Seokwon Kim, Dahye Lee, Hanseung Baek, Bae-Kyung Kim, Young-Youn Kim, Soo-Bok Her, Dong-Yub Ko, Inseong Hwang, Hye-Sung Kim

**Affiliations:** 1 Apple Tree Institute of Biomedical Science, Apple Tree Medical Foundation, Goyang, Republic of Korea; 2 Apple Tree Dental Hospital, Apple Tree Medical Foundation, Goyang, Republic of Korea; 3 Research and Development Center, DOCSMEDI Co. Ltd., Goyang, Republic of Korea; 4 Since 1982 THE Lee Hae-seung Dental Clinic, Paju-si, Gyeonggi-do, Republic of Korea; 5 Artificial Intelligence Research Center, Digital Dental Hub Incorporation, Seoul, Republic of Korea; 6 COREE Pohang Co., Ltd., Pohang-si, Gyeongsangbuk-do, Republic of Korea

**Keywords:** absolute quantification, mouthwash, periodontal microbiome, periodontitis, *Porphyromonas gingivalis*, quantitative real-time PCR, treatment monitoring

## Abstract

**Background:**

Non-invasive molecular tools that can both screen for severe periodontitis and quantify treatment-associated pathogen changes remain an unmet need. We evaluated a non-invasive multiplex quantitative polymerase chain reaction (qPCR) mouthwash panel providing absolute colony-forming-unit (CFU) quantification of five periodontal pathogens (*Porphyromonas gingivalis, Treponema denticola, Tannerella forsythia, Prevotella intermedia, Fusobacterium nucleatum*), and asked whether a single test can both screen for severe disease and track each patient’s species-level treatment response.

**Methods:**

Treatment-response monitoring was assessed in a real-world retrospective cohort (349 patients, 703 visits; 235 paired pre/post-treatment samples) using paired Wilcoxon signed-rank tests and Jonckheere–Terpstra trend tests across baseline stage. Screening performance was independently evaluated in a prospective cross-sectional cohort (n=87; 31 healthy, 56 Stage III/IV) with leave-one-out cross-validation (LOOCV) and a pre-specified sensitivity analysis.

**Results:**

For screening, LOOCV AUC was 0.96 for *P. gingivalis* and 0.97 for the combined panel, preserved under inclusion of antibiotic- and treatment-exposed subjects. Beyond screening, the panel resolved each patient’s species-level response to non-surgical therapy: all five species decreased significantly, with median within-patient reductions of −0.37 to −0.72 log_10_ CFU (paired Wilcoxon, all p<0.001) and reduction magnitude scaling with baseline severity. This response was strongly species-specific and clinically informative at the individual level: In the implant-bearing high-baseline-burden subgroup, post-treatment *F. nucleatum* reached or fell below the Healthy reference median in 61% of evaluable patients, whereas *P. gingivalis* reached it in only 5% and remained approximately 500-fold above the Healthy reference at the median final visit, identifying residual keystone-pathogen burden not captured by clinical or radiographic assessment.

**Conclusion:**

In this two-cohort evaluation, a single non-invasive mouthwash qPCR test provided two linked clinical outputs: high-accuracy screening for severe periodontitis and quantitative individual-level tracking of species-specific microbial response to therapy. These findings are preliminary, derive from a single-center screening cohort and a retrospective monitoring cohort, and require external validation before clinical deployment. By placing pathogen loads on the same absolute CFU scale, the assay supports both cross-sectional disease detection and longitudinal residual-burden monitoring as an adjunct to the 2018 AAP/EFP framework. Clinical Research Information Service (CRIS) registration: KCT0011511 (https://cris.nih.go.kr).

## 1 Introduction

Periodontitis affects approximately half of the global adult population and is the leading cause of tooth loss in adults (Wu et al., 2025). Under the 2018 American Academy of Periodontology/European Federation of Periodontology (AAP/EFP) classification framework, diagnosis and staging rely on clinical attachment loss (CAL), radiographic bone loss, and the pattern of tissue destruction (Tonetti et al., 2018). This framework provides robust criteria for definitive diagnosis, but two practical gaps remain. First, a simple non-invasive screening or triage tool could help identify individuals likely to have severe periodontitis when full periodontal charting is not immediately available. Second, current clinical and radiographic measures do not show what happens microbiologically inside an individual patient after treatment: they can confirm whether attachment and bone have stabilized, but cannot indicate which pathogenic species have been cleared, which persist, or how much residual microbial burden remains from visit to visit (Papapanou et al., 2018).

This monitoring gap is clinically consequential. Non-surgical periodontal therapy reduces total bacterial load, but species differ in their susceptibility to mechanical disruption and in their capacity to repopulate protected niches; a patient judged clinically improved may still harbor a substantial residual burden of keystone pathogens (Iniesta et al., 2024) (Krajewski et al., 2025). Yet the methods that could reveal this—subgingival plaque sampling with culture or sequencing—are impractical for repeated longitudinal use. A non-invasive test quantifying pre-specified pathogens with sufficient precision to resolve each patient’s species-level response over time would therefore address a need the diagnostic framework, however robust, leaves open (Kim et al., 2022).

The microbial etiology of periodontitis has evolved over three decades from the red-complex paradigm (Socransky et al., 1998) to the polymicrobial synergy and dysbiosis model that integrates established pathogens with emerging pathobionts and ecologically supportive species (Hajishengallis et al., 2023). Mouthwash sampling offers a validated non-invasive alternative to subgingival sampling, and qPCR-based absolute quantification of pre-specified targets could combine operational simplicity with the quantitative precision required for longitudinal monitoring (Belstrøm et al., 2017).

We evaluated a multiplex qPCR panel delivering absolute colony-forming-unit (CFU) quantification of five periodontal pathogens—*Porphyromonas gingivalis*, *Treponema denticola*, *Tannerella forsythia, Prevotella intermedia*, and *Fusobacterium nucleatum*—on a standard-curve system previously validated against shotgun metagenomic sequencing (Hwang et al., 2024). The five-pathogen composition was selected *a priori* to integrate the three red-complex species with the orange complex (*P. intermedia*) and the bridge organism (*F. nucleatum*), reflecting the polymicrobial framework. A complementary 16S rRNA sequencing analysis of community-level taxonomic structure in the same enrollment population has been reported separately and is non-overlapping with the targeted quantification outcomes presented here. Our central question was whether a single non-invasive absolute-CFU mouthwash test could support two complementary clinical roles: high-accuracy screening for severe periodontitis and longitudinal quantification of each patient’s species-level microbial response to therapy (Fan et al., 2023). We therefore evaluated both cross-sectional screening performance for discriminating Stage III–IV periodontitis from health and paired real-world treatment-monitoring utility for quantifying pre- and post-treatment microbial change on the same absolute CFU scale. The panel is positioned as a non-invasive adjunct intended to complement, not replace, current AAP/EFP diagnostic standards.

## 2 Materials and methods

### 2.1 Study design and ethics

This work comprises two non-overlapping analyses: a prospective cross-sectional cohort for screening performance (Stage III–IV periodontitis) and a retrospective audit cohort for longitudinal treatment-monitoring utility. Reporting follows STARD 2015 (Bossuyt et al., 2015), MIQE 2009 (Bustin et al., 2009, 2025), and STROBE (Von Elm et al., 2007). The cross-sectional cohort was approved by the Apple Tree Medical Foundation IRB (ATDH-2025-0001) and registered (Clinical Research Information Service [CRIS] KCT0011511; retrospective registration 21 January 2026); written informed consent was obtained. The study was conducted in accordance with the Declaration of Helsinki. The audit cohort was approved separately (ATDH-2026-0003) with consent waived for retrospective review of de-identified records.

### 2.2 Study cohorts

Two independent cohorts were analyzed under the 2018 AAP/EFP framework (Tonetti et al., 2018), with staging based on interdental CAL, radiographic bone loss, and tooth loss. Cohort 1 (cross-sectional) comprised adults aged ≥19 years prospectively enrolled at Apple Tree Dental Hospital (Goyang, Republic of Korea; February 2025–March 2026), under a shared protocol with a companion 16S rRNA study (Cho et al., 2026); outcomes are non-overlapping. Healthy status required probing depth ≤3 mm, bleeding on probing <10%, and no radiographic bone loss; exclusions encompassed recent (≤3 months) anti-inflammatory, immunosuppressive, or antimicrobial use, prior periodontal treatment, uncontrolled systemic disease, pregnancy, oral malignancy, or substance abuse. Of 134 screened, 25 were excluded (antibiotics n=13, periodontal treatment n=5, both n=3, missing PD/BOP n=4), leaving 109 included; the primary analytic subset was 87 (Healthy n=31; Stage III n=17; Stage IV n=39), with intermediate stages (Gingivitis n=5; Stage I/II n=17) retained for ordinal trend analyses only, and antibiotic- or treatment-exposed subjects (n=21) re-introduced for sensitivity analysis. Full-mouth six-site examination recorded probing depth (PD), bleeding on probing (BOP), and remaining teeth.

Cohort 2 (longitudinal real-world audit) comprised patients who underwent the OBCheck mouthwash multiplex qPCR test (October 2023–April 2026; IRB ATDH-2026-0003, consent waived); of 374 patients identified, 349 had a baseline panoramic radiograph permitting estimated CAL (eCAL) and contributed 703 visits (range 1–6 per patient; follow-up at V2 n=242, V3 n=104), with 235 patients in a paired pre/post-treatment sub-cohort (median interval 395 days, IQR 248–546). This repeated-sampling structure—multiple visits per patient over a clinically realistic interval—enabled the second major clinical objective of the study: within-patient, species-level treatment tracking on the same absolute-CFU scale used for screening. For both cohorts, panoramic radiographs were analyzed using a deep-learning platform (PANO; Digital Dental Hub Incorporation, Republic of Korea) to derive eCAL by subtracting 2.04 mm biological width from interdental crestal bone level (Gargiulo et al., 1961; Chang et al., 2020); per-subject eCAL (maximum across measurable sites) was classified using a three-category framework—Healthy/Gingivitis (≤2 mm), Stage I–II (2–4 mm), and Severe Stage III–IV (>4 mm)—aligning with the primary anchor analysis of severe versus non-severe disease (**Supplementary Figure 4**).

### 2.3 Mouthwash sample collection and DNA extraction

After standardized tooth brushing, each participant rinsed 10 mL of COOL SENSE Stick Gargle (Docsmedi Co., Ltd., Republic of Korea) for 30 seconds and expectorated into a sterile polypropylene tube; samples were stored at −80 °C until DNA extraction. From each gargle sample, 500 µL was centrifuged at 10,000 rpm for 5 minutes at room temperature, the supernatant discarded, and the cell pellet retained for extraction. DNA was extracted on an ARA-MAG MultiEx 032 automated nucleic-acid extraction system (LAS Co., Ltd., Republic of Korea) with the ARA MagNa Universal Isolation Kit according to the manufacturer’s instructions, eluted in 50 µL of elution buffer, and stored at −20 °C until qPCR analysis.

### 2.4 Multiplex qPCR quantification

Absolute pathogen loads were measured by multiplex real-time qPCR (OBCheck; Docsmedi Co., Ltd.) using species-specific TaqMan primer–probe sets. Each 20 µL reaction contained 10 µL of 2× PCR Master Mix (Enzynomics or Bioneer, Republic of Korea), 1 µL template DNA, primers at 200 nM, and probes at 100 nM final concentration, organized into multiplex sets to minimize fluorescence-channel interference. Amplification was performed on a QuantStudio 3 Real-Time PCR System (Thermo Fisher Scientific, Waltham, MA, USA) under the following cycling protocol: initial denaturation at 95 °C for 10 min, followed by 40 cycles of 95 °C for 15 s, 55 °C for 30 s, and 72 °C for 30 s. A threshold cycle (Ct) exceeding 40 was designated negative (CFU = 0). Rather than re-establishing a full standard curve within each analytical run, quantification used the pre-established, lot-specific species-specific standard curves generated and validated in a dedicated methodological study (Hwang et al., 2024); every run included no-template controls and positive amplification controls, and runs were accepted only when these controls performed as expected, so that run-to-run performance was monitored against the controls rather than by repeated curve construction. Absolute CFU/mL was derived by interpolation against these species-specific standard curves, in which the CFU–Ct standard curves were linear across the quantification range (coefficient of determination r² > 0.99 for all species), predicted CFU accuracy was confirmed by a spiking recovery study (recovery 95.1–106.8%), and quantification was cross-validated against shotgun metagenomic sequencing (Spearman ρ = 1.0); this absolute, standard-curve–based quantification yields a value on a fixed scale for every sample, allowing CFU loads to be compared directly across visits within a patient without the relative-abundance normalization required by sequencing-based methods. Full primer and probe target genes and sequences are provided in **Supplementary Table 3**. Laboratory analysts were blinded to clinical status and clinicians to qPCR results. The red complex composite was defined as log_10_ of the summed CFU/mL across *P. gingivalis, T. denticola, and T. forsythia.*

### 2.5 Outcomes

Primary outcomes were (i) discrimination of Stage III–IV vs Healthy by single-species and combined five-pathogen leave-one-out cross-validation (LOOCV) area under the curve (AUC), and (ii) paired pre/post-treatment change in log_10_ CFU. Secondary outcomes were monotonic trend across the four-group severity gradient, age- and sex-adjusted between-group differences, Spearman correlation with periodontal parameters, and sensitivity to inclusion of subjects with antibiotic or periodontal-treatment exposure.

### 2.6 Statistical analysis

Analyses used Python 3.13 (SciPy, statsmodels, scikit-learn; two-sided α = 0.05). Monotonic association of log_10_ CFU with the four-group ordinal severity gradient was assessed by the Jonckheere–Terpstra trend test. Logistic regression for Stage III–IV vs Healthy was fitted for each single species and for the five-species combined panel, with log_10_ CFU values entered as forced predictors without variable selection; AUC was reported with 2,000-iteration bootstrap 95% CI, and LOOCV with optimism correction was used as the primary internal validation method (Steyerberg et al., 2010). Single-species cutoffs were derived by the Youden index applied directly to log_10_ CFU; clinical utility was assessed by decision curve analysis (Vickers et al., 2016). ANCOVA estimated age- and sex-adjusted between-group differences, with Benjamini–Hochberg FDR control across five species. Between-group differences in log_10_ CFU across the four severity groups were assessed by the Kruskal–Wallis test, with *post-hoc* pairwise comparisons by Dunn’s test and Benjamini–Hochberg correction.

For the longitudinal sub-cohort, within-subject change in log_10_ CFU was tested by paired Wilcoxon signed-rank test with median Δlog_10_ and 2,000-iteration bootstrap 95% CI; the Jonckheere–Terpstra test assessed trend in treatment effect across baseline stage. Because the monitoring analysis is paired within patient, each subject serves as their own baseline, removing between-subject variability and allowing species-specific change to be resolved at the individual level. A pre-specified sensitivity analysis re-introduced 8 exposure-excluded subjects who otherwise qualified for the primary anchor groups, yielding n=95 (Healthy n=32; Stage III–IV n=63), and all primary screening analyses were repeated under this composition. Below-limit-of-detection (BLOD) values were treated as missing in single-species analyses and imputed at one-half the species-specific limit of detection in combined analyses, following MIQE recommendations for handling values below the reliable quantification limit. To confirm that treatment-response conclusions did not depend on this choice, a pre-specified sensitivity analysis re-computed all paired within-patient changes under three BLOD rules—excluded as missing, imputed at one-half LOD, and floored at 1 CFU—on the identical cohort (**Supplementary Table 4**; **Supplementary Figure 9**). In Cohort 1 screening analyses, per-species BLOD counts (*P. gingivalis/T. denticola/T. forsythia/P. intermedia/F. nucleatum*) were 15/10/8/28/2, leaving evaluable single-species counts of 72/77/79/59/85, respectively.

## 3 Results

### 3.1 Participant characteristics

The cross-sectional cohort comprised 87 participants in the two anchor groups (Healthy n=31; Stage III–IV n=56), with 22 intermediate-stage subjects (Gingivitis n=5; Stage I/II n=17) retained for ordinal trend analyses. Demographic and clinical characteristics across the four severity groups are summarized in **Table 1**; mean age, PD, BOP, eCAL, and proportion of remaining teeth all worsened monotonically with stage.

**Table 1:** Demographic and clinical characteristics of the cross-sectional cohort by AAP/EFP severity group (n = 109).

Characteristic	Healthy (n = 31)	Intermediate (n = 22)	Stage III (n = 17)	Stage IV (n = 39)	p-value
Age, years (mean ± SD)	31.4 ± 6.3	43.8 ± 13.4	50.5 ± 16.7	57.8 ± 15.8	<0.001
Female, n (%)	27 (87.1)	9 (40.9)	10 (58.8)	18 (46.2)	0.001
Remaining teeth (mean ± SD)	27.8 ± 0.9	27.2 ± 2.0	25.9 ± 2.4	16.6 ± 6.2	<0.001
Mean probing depth, mm (mean ± SD)	2.20 ± 0.50	2.65 ± 0.54	3.37 ± 0.59	3.47 ± 1.27	<0.001
Maximum probing depth, mm (mean ± SD)	3.0 ± 0.0	4.4 ± 1.0	8.9 ± 1.0	8.4 ± 2.1	<0.001
Bleeding on probing, % (mean ± SD)	3.7 ± 2.9	18.8 ± 9.9	35.8 ± 27.3	32.5 ± 31.8	<0.001
Mean eCAL, mm (mean ± SD)	0.36 ± 0.20	0.78 ± 0.53	2.47 ± 0.42	2.97 ± 0.74	<0.001
Maximum eCAL, mm (mean ± SD)	1.9 ± 0.5	2.5 ± 1.0	7.8 ± 1.8	7.0 ± 1.7	<0.001
Absolute CFU load, median (interquartile range)					
*P. gingivalis*, CFU/mL	2.1×10² (3.5×10¹–9.6×10³) ^a^	4.6×10^4^ (1.9×10^4^–1.8×10^5^) ^a^	2.4×10^6^ (8.1×10^5^–3.3×10^6^) ^b^	1.2×10^6^ (3.2×10^5^–3.7×10^6^) ^b^	<0.001
*T. denticola*, CFU/mL	4.0×10³ (9.2×10²–3.3×10^4^) ^a^	2.6×10^4^ (1.3×10^4^–5.1×10^4^) ^a^	3.1×10^5^ (1.9×10^5^–6.9×10^5^) ^b^	4.4×10^5^ (1.4×10^5^–7.4×10^5^) ^b^	<0.001
*T. forsythia*, CFU/mL	5.6×10³ (9.2×10²–8.7×10³) ^a^	1.4×10^4^ (6.3×10³–2.9×10^4^) ^a^	9.7×10^4^ (3.8×10^4^–2.0×10^5^) ^b^	9.0×10^4^ (3.3×10^4^–2.0×10^5^) ^b^	<0.001
*P. intermedia*, CFU/mL	1.0×10^4^ (4.2×10³–5.7×10^4^) ^a^	3.9×10^4^ (5.9×10³–1.1×10^5^) ^ab^	3.0×10^5^ (8.7×10^4^–6.0×10^5^) ^b^	1.4×10^5^ (5.5×10^4^–2.6×10^5^) ^b^	0.001
*F. nucleatum*, CFU/mL	6.3×10^4^ (3.6×10^4^–1.0×10^5^) ^a^	1.2×10^5^ (6.4×10^4^–1.9×10^5^) ^ab^	4.1×10^5^ (1.9×10^5^–7.0×10^5^) ^b^	2.0×10^5^ (9.4×10^4^–3.6×10^5^) ^b^	<0.001

eCAL, estimated clinical attachment loss derived from panoramic AI-measured interdental crestal bone level minus 2.04 mm biological width. CFU, colony-forming units. P-values: one-way ANOVA for continuous variables, chi-square test for categorical sex distribution, Kruskal–Wallis test across four ordered groups for CFU loads, with pairwise *post-hoc* comparisons by Dunn’s test (Benjamini–Hochberg-adjusted). For each species, medians not sharing a common superscript letter differ significantly (adjusted p < 0.05); all significant differences were between Stage III/IV and the Healthy/Intermediate groups, with no significant difference between Stage III and Stage IV, nor between Healthy and Intermediate, for any species. The “Intermediate” group combines Gingivitis (n = 5) and Stage I/II (n = 17) subjects retained for ordinal trend analyses but excluded from binary screening evaluation (primary cohort n = 87: Healthy + Stage III + Stage IV). Several site-based measures (maximum probing depth, maximum eCAL, bleeding on probing) and some CFU medians are marginally lower in Stage IV than Stage III, reflecting the substantially reduced number of remaining teeth and measurable sites in Stage IV (mean 16.6 vs 25.9 teeth) rather than lower disease severity. Under the 2018 AAP/EFP framework, tooth loss due to periodontitis is itself a staging criterion (Stage III, ≤4 teeth; Stage IV, ≥5 teeth); the whole-mouth mouthwash sample therefore integrates fewer colonized sites at higher stage, which lowers absolute CFU per sample without altering the monotonic increase in per-site microbial burden across the severity gradient.

Internal validation of AI-derived eCAL staging against clinician-assigned stage was performed in all 109 Cohort 1 subjects (Healthy/Gingivitis n=36; Stage I–II n=17; Severe Stage III–IV n=56). Exact agreement was 76.1%, adjacent (± 1 stage) agreement was 100% with no subject misclassified by more than one category, Cohen’s linear-weighted κ = 0.74 (substantial) and quadratic-weighted κ = 0.84 (almost perfect), and eCAL-based assignment showed strong monotonic association with clinician stage (Jonckheere–Terpstra z = +9.56, p < 0.001; **Supplementary Figure 4**). For the principal binary classification of severe (Stage III–IV) versus non-severe disease, eCAL achieved 94.6% sensitivity (53/56, 95% CI 85.4–98.2%) and 96.2% specificity (51/53, 95% CI 87.2–99.0%), supporting its use for stage assignment in Cohort 2, where direct clinical measurements were unavailable, and confirming that eCAL preserves the ordinal severity rank required for the stage-dependent analyses that follow.

The longitudinal audit cohort comprised 349 patients contributing 703 visits, of whom 235 had documented paired pre/post-treatment samples following scaling or scaling-and-root-planing (median interval 395 days, IQR 248–546 days; baseline characteristics in **Supplementary Figure 8**).

### 3.2 Absolute CFU loads increased monotonically with periodontal stage

**Figure 1:**
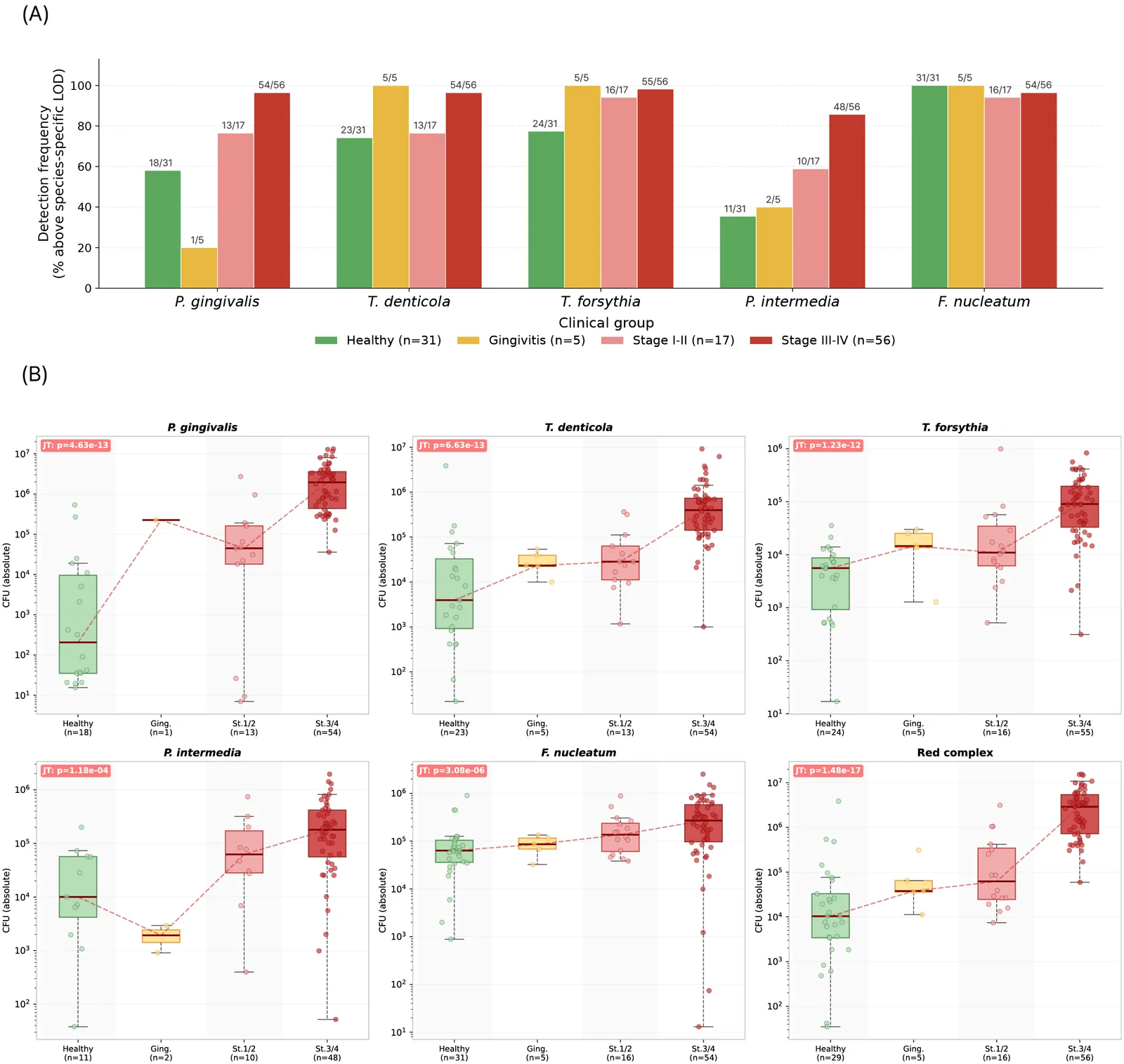
Detection frequency and absolute CFU loads of five periodontal pathogens across the four-group periodontal severity gradient (cross-sectional cohort). **(A)** Detection frequency, defined as the percentage of subjects in each clinical group with a signal above the species-specific limit of detection (LOD); numerators and denominators (subjects detected/group total) are printed above each bar. Denominators are full clinical-group sizes: Healthy n=31, Gingivitis n=5, Stage I–II n=17, Stage III–IV n=56. Detection frequency of the three red-complex species (*P. gingivalis, T. denticola, T. forsythia*) and of *P. intermedia* rises with clinical severity, whereas *F. nucleatum* is detected in nearly all subjects irrespective of group, consistent with its interpretation as an ecologically supportive co-marker. **(B)** Boxplots of absolute CFU loads on a log10 y-axis for *Porphyromonas gingivalis*, *Treponema denticola*, *Tannerella forsythia*, *Prevotella intermedia*, *Fusobacterium nucleatum*, and the red complex composite, stratified by clinician-assigned status: Healthy, Gingivitis, Stage I/II, and Stage III/IV. Boxes show the interquartile range with the median as a dark line; whiskers extend to 1.5 × IQR, with subjects overlaid as jittered points. Dashed red lines connect group medians. In **(B)**, per-panel n is the evaluable count after BLOD exclusion (shown on each panel) and therefore differs from the full group sizes in **(A)** Inset boxes report Jonckheere–Terpstra (JT) trend p-values. CFU, colony-forming units; LOD, limit of detection; JT, Jonckheere–Terpstra trend test.

Detection frequency above the species-specific limit of detection rose with clinical severity for the three red-complex species and for *P. intermedia*, whereas *F. nucleatum* was detected in nearly all subjects across every group (**Figure 1A**). Beyond mere detection, absolute CFU loads of all three red-complex species increased monotonically across the four ordered clinical groups (**Figure 1B**), with Jonckheere–Terpstra trend p ≤ 1.2 × 10^−^¹² for *P. gingivalis*, *T. denticola*, and *T. forsythia*, and p = 1.5 × 10^−17^ for the red complex composite.

### 3.3 CFU loads correlated with clinical periodontal parameters

**Figure 2:**
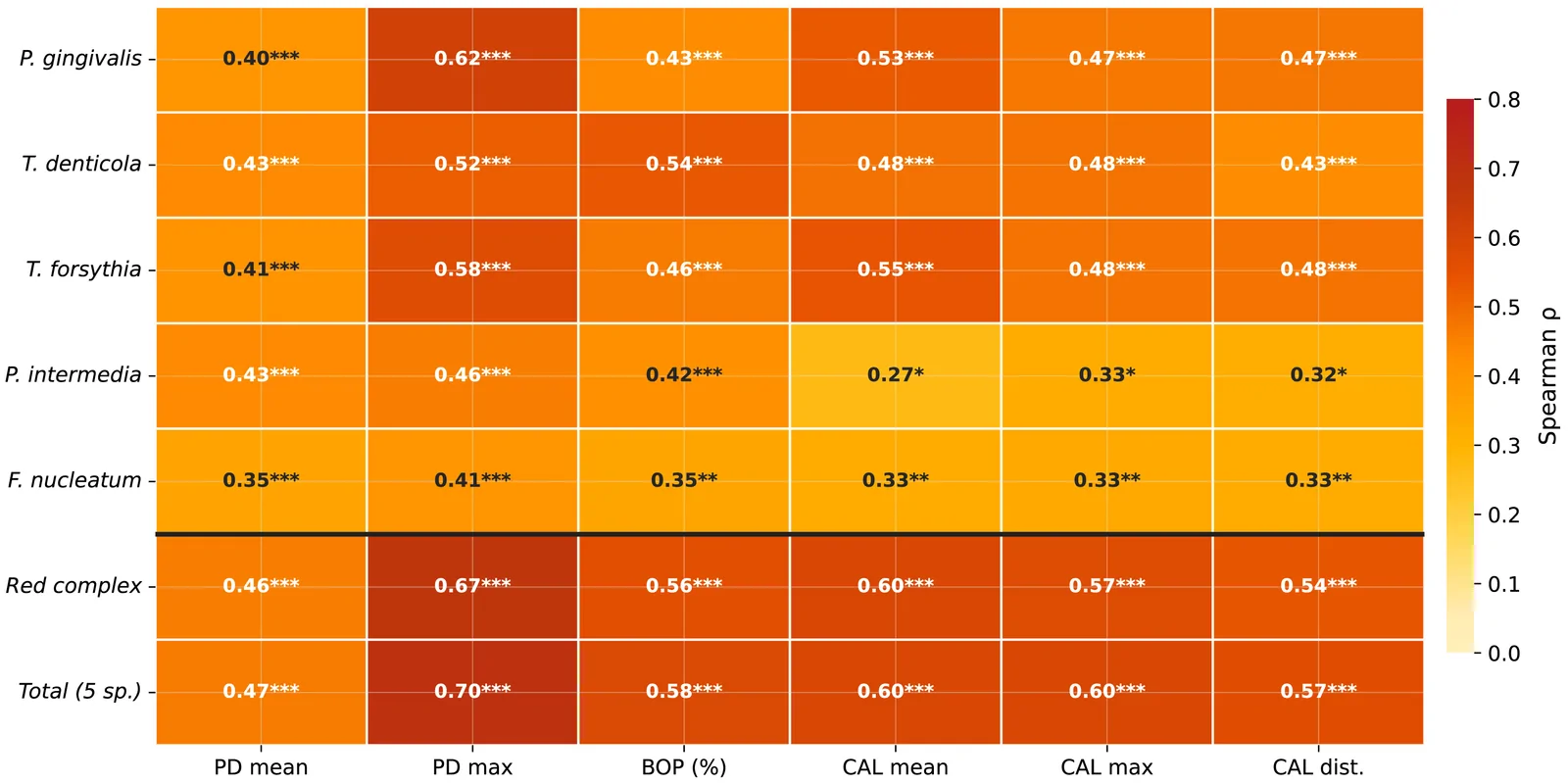
Spearman rank correlations between absolute CFU loads and clinical periodontal parameters (n=87). Heatmap of Spearman correlation coefficients (ρ) between log10 CFU of each pathogen (rows: five individual species; red complex composite; total five-species sum) and six clinical periodontal parameters (columns: mean and maximum probing depth, percentage of sites with bleeding on probing, mean and maximum AI-derived estimated clinical attachment loss, and CAL distribution score). Cell color encodes ρ on a yellow-to-red gradient; the numeric ρ value and significance level are overlaid in each cell. Horizontal bold line separates single-species rows from composite scores. Significance levels: ***p < 0.001; **p < 0.01; *p < 0.05. AI, artificial intelligence; BOP, bleeding on probing; CAL, clinical attachment loss; CFU, colony-forming units; PD, probing depth.

CFU loads were significantly associated with all measured clinical parameters (PD, BOP, eCAL; **Figure 2**). The five-species total and red complex composite showed the strongest correlations with maximum PD (Spearman ρ = 0.70 and 0.67 respectively; p < 0.001), and ρ ≥ 0.58 with BOP and eCAL. *P. gingivalis* alone reached ρ = 0.62 with maximum PD (p < 0.001); *P. intermedia* and *F. nucleatum* showed weaker but consistently significant correlations (ρ = 0.27–0.46).

### 3.4 Five-pathogen panel discriminates severe periodontitis with high screening performance

**Figure 3:**
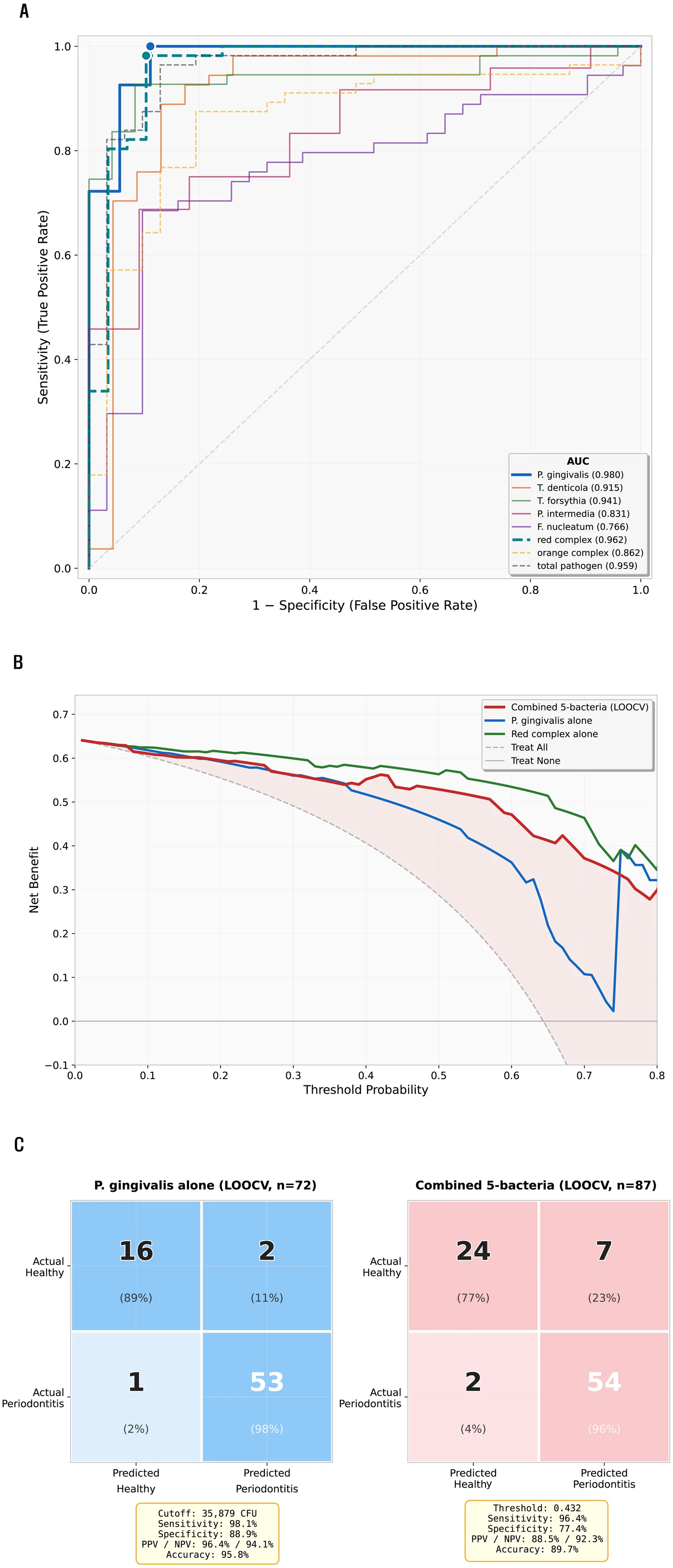
Screening performance of single-species and combined five-pathogen models for Stage III–IV periodontitis versus health (cross-sectional cohort). **(A)** ROC curves for the five single species, the red complex composite, the orange complex (*P. intermedia* + *F. nucleatum*), and the five-species total pathogen score, with AUC values in the legend. The two filled markers indicate the Youden-optimal operating points for *P. gingivalis* alone (solid blue) and the combined five-species LOOCV model (dashed teal). **(B)** Decision curve analysis showing net benefit across the clinically relevant range of threshold probabilities for the combined five-bacteria LOOCV model (red), *P. gingivalis* alone (blue), and the red complex alone (green), referenced against treat-all and treat-none strategies. **(C)** Confusion matrices under LOOCV for *P. gingivalis* alone (left, evaluable n=72; cutoff 35,879 CFU) and the combined five-bacteria logistic-regression model (right, n=87; probability threshold 0.432). Inset boxes report sensitivity, specificity, PPV, NPV, and accuracy. AUC, area under the curve; LOOCV, leave-one-out cross-validation; NPV, negative predictive value; PPV, positive predictive value.

ROC analysis demonstrated strong discriminative performance (**Figure 3A**; AUC and 95% CI summarized in **Supplementary Table 2**). *P. gingivalis* alone achieved the highest single-species discrimination (raw ROC AUC 0.98, 95% CI 0.94–1.00; LOOCV AUC 0.96, 95% CI 0.91–1.00; optimism 0.02; evaluable n=72). The red complex composite reached AUC 0.98 (95% CI 0.96–1.00), and the combined five-species logistic regression model achieved apparent AUC 0.99 (95% CI 0.96–1.00) with LOOCV AUC 0.97 (95% CI 0.92–1.00; optimism 0.02; n=87). *T. denticola* (AUC 0.92, 95% CI 0.81–0.99) and *T. forsythia* (0.94, 0.88–0.99) also achieved high single-species discrimination, while *F. nucleatum* (0.77, 0.65–0.86) and *P. intermedia* (0.83, 0.70–0.94) confirmed their secondary role as ecologically supportive co-markers.

At the Youden-optimal threshold derived from raw log_10_ CFU, *P. gingivalis* alone (cutoff 35,879 CFU) yielded sensitivity 98.1%, specificity 88.9%, positive predictive value (PPV) 96.4%, negative predictive value (NPV) 94.1%, and accuracy 95.8% (**Figure 3C**, left). The combined five-species model at LOOCV probability threshold 0.432 yielded sensitivity 96.4%, specificity 77.4%, PPV 88.5%, NPV 92.3%, and accuracy 89.7% (**Figure 3C**, right). Decision curve analysis showed that, across the clinically relevant range of threshold probabilities (approximately 0.1–0.6), using the panel to decide who warrants full periodontal assessment yielded greater net benefit than either referring all patients (treat-all) or referring none (treat-none)—the two default strategies in the absence of a triage test—indicating a net gain in correctly identified severe cases without a corresponding excess of unnecessary referrals. The red complex composite alone provided net benefit comparable to or exceeding the full combined model over much of this range, indicating that a three-species red-complex readout captures most of the screening signal (**Figure 3B**; balanced-criterion cutoff verification in **Supplementary Figure 5**).

### 3.5 Screening performance was robust after adjustment for age and sex

After ANCOVA adjustment for age and sex (**Supplementary Table 1**; **Supplementary Figure 1**), the between-group difference (Stage III–IV vs Healthy) remained large and highly significant for the three red-complex species and for the red complex composite (β = +3.61 log_10_ CFU for *P. gingivalis*, p = 2.8 × 10^−18^, R² = 0.74; β = +1.86 for *T. denticola*, p = 3.0 × 10^−9^; β = +1.31 for *T. forsythia*, p = 5.2 × 10^−8^; β = +2.51 for the red complex composite, R² = 0.67; all FDR q ≤ 1.7 × 10^−7^). *P. intermedia* retained a weaker but significant effect (β = +1.26, p = 8.0 × 10^−4^); *F. nucleatum* attenuated to non-significance (β = −0.08, p = 0.74), consistent with its role as an ecological co-marker (partial correlations, **Supplementary Figure 2**). Sex was not an independent predictor (all p > 0.17). In a multivariable logistic-regression model containing all five species (each log_10_ CFU standardized to unit SD), a 1 SD increase retained a significant independent association with severe periodontitis for the three red-complex species (**Supplementary Figure 7**): *P. gingivalis* OR 3.68 (95% CI 2.08–6.11), *T. denticola* OR 3.48 (2.04–6.41), and *T. forsythia* OR 2.84 (1.65–6.25). After mutual adjustment, *F. nucleatum* (OR 1.46, 0.91–2.49) and *P. intermedia* (OR 0.94, 0.47–1.38) were not independently significant, consistent with their role as ecologically supportive co-markers.

### 3.6 Pre-specified sensitivity analysis confirms robustness under real-world exposure conditions

Re-introducing 8 subjects whose clinical profiles would have qualified for the primary cohort’s anchor groups in the absence of antibiotic or periodontal-treatment exposure exclusion yielded n=95 (Healthy n=32; Stage III–IV n=63; **Supplementary Figure 6**). Combined five-species LOOCV AUC was 0.93 (95% CI 0.87–0.97; apparent AUC 0.95; optimism 0.02). Single-species sensitivity-cohort AUCs were 0.97 for *P. gingivalis* (95% CI 0.93–1.00), 0.94 for *T. forsythia* (0.89–0.99), and 0.91 for *T. denticola* (0.82–0.98). Screening performance was therefore preserved under inclusion of subjects with antibiotic and/or periodontal-treatment exposure histories, supporting robustness against deployment-related selection effects.

### 3.7 The panel quantifies each patient’s species-level response to therapy

**Figure 4:**
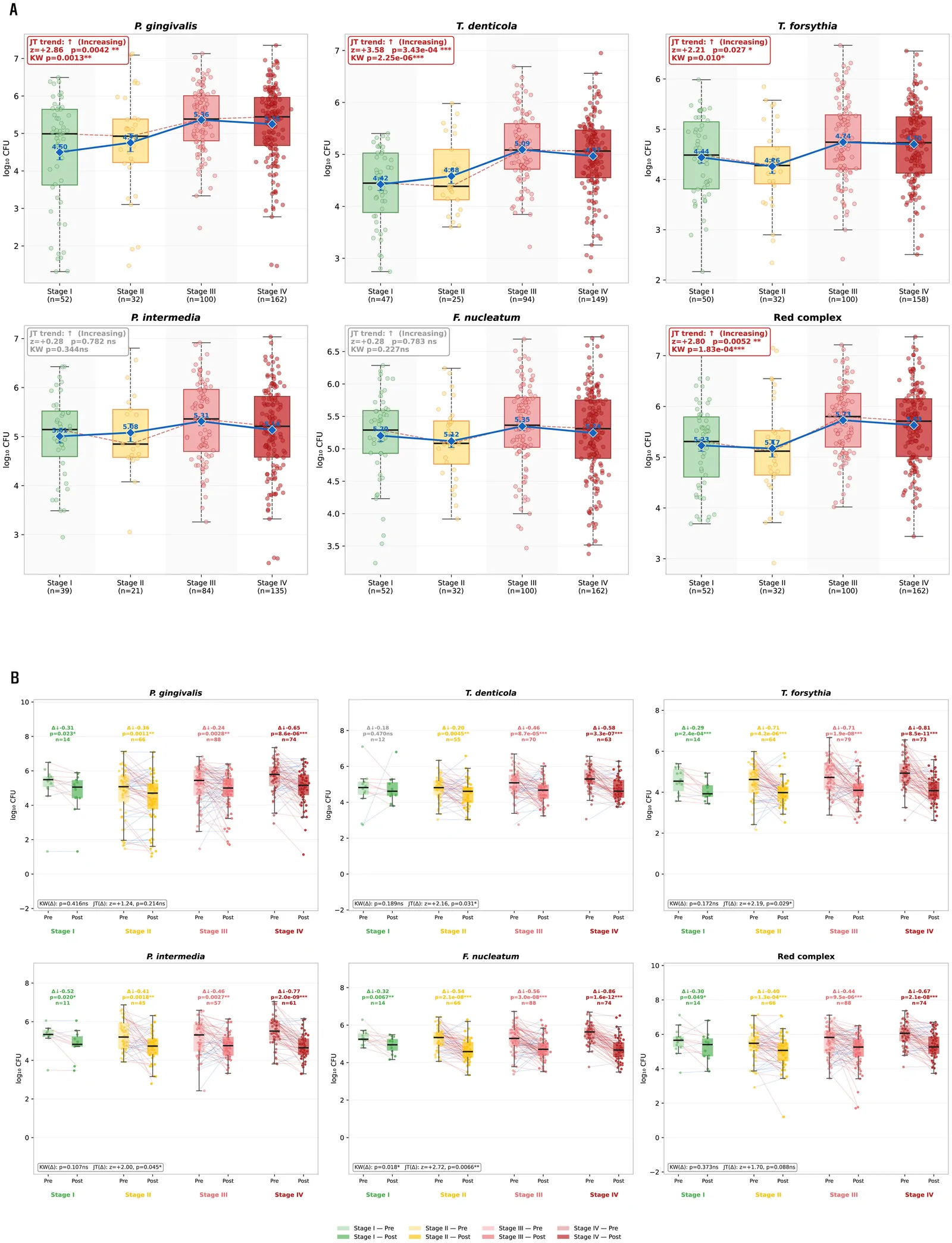
Longitudinal CFU trajectories and stage-stratified treatment response in the real-world audit cohort. **(A)** Baseline (Visit 1) log10 CFU distributions of the five species and the red complex composite, stratified by baseline AAP/EFP stage. Boxplots show the interquartile range with subjects overlaid; superimposed blue diamonds and connecting lines indicate group means. Inset boxes report Jonckheere–Terpstra (JT) trend statistics and the Kruskal–Wallis (KW) omnibus p-value. **(B)** Paired pre/post-treatment log10 CFU within each baseline-stage stratum following documented scaling or scaling-and-root-planing. Annotation boxes report the within-stratum median Δlog10 (Post − Pre) with paired Wilcoxon p-value; KW(Δ) and JT(Δ) tests for trend in treatment effect across baseline stages are shown in the lower bar. CFU, colony-forming units; JT, Jonckheere–Terpstra; KW, Kruskal–Wallis.

Using the same absolute-CFU scale that supported cross-sectional screening, the panel also resolved within-patient microbial change after treatment. In the longitudinal cohort, all five pathogens showed statistically significant within-subject CFU reductions after documented scaling or scaling-and-root-planing (n=235 paired samples; **Figure 4**; **Supplementary Figure 3**). Median Δlog_10_ CFU was −0.37 for P. gingivalis, −0.44 for T. denticola, −0.72 for T. forsythia, −0.53 for P. intermedia, −0.66 for F. nucleatum, and −0.45 for the red complex composite (all paired Wilcoxon p ≤ 1.8 × 10^−10^; all significant after Benjamini–Hochberg correction). These reductions were invariant to below-limit-of-detection handling: median Δlog_10_ and significance were essentially unchanged across BLOD-excluded, one-half-LOD, and 1-CFU-floor rules, with only the red complex composite shifting trivially (−0.445 to −0.452; **Supplementary Table 4**; **Supplementary Figure 9**).

**Figure 5:**
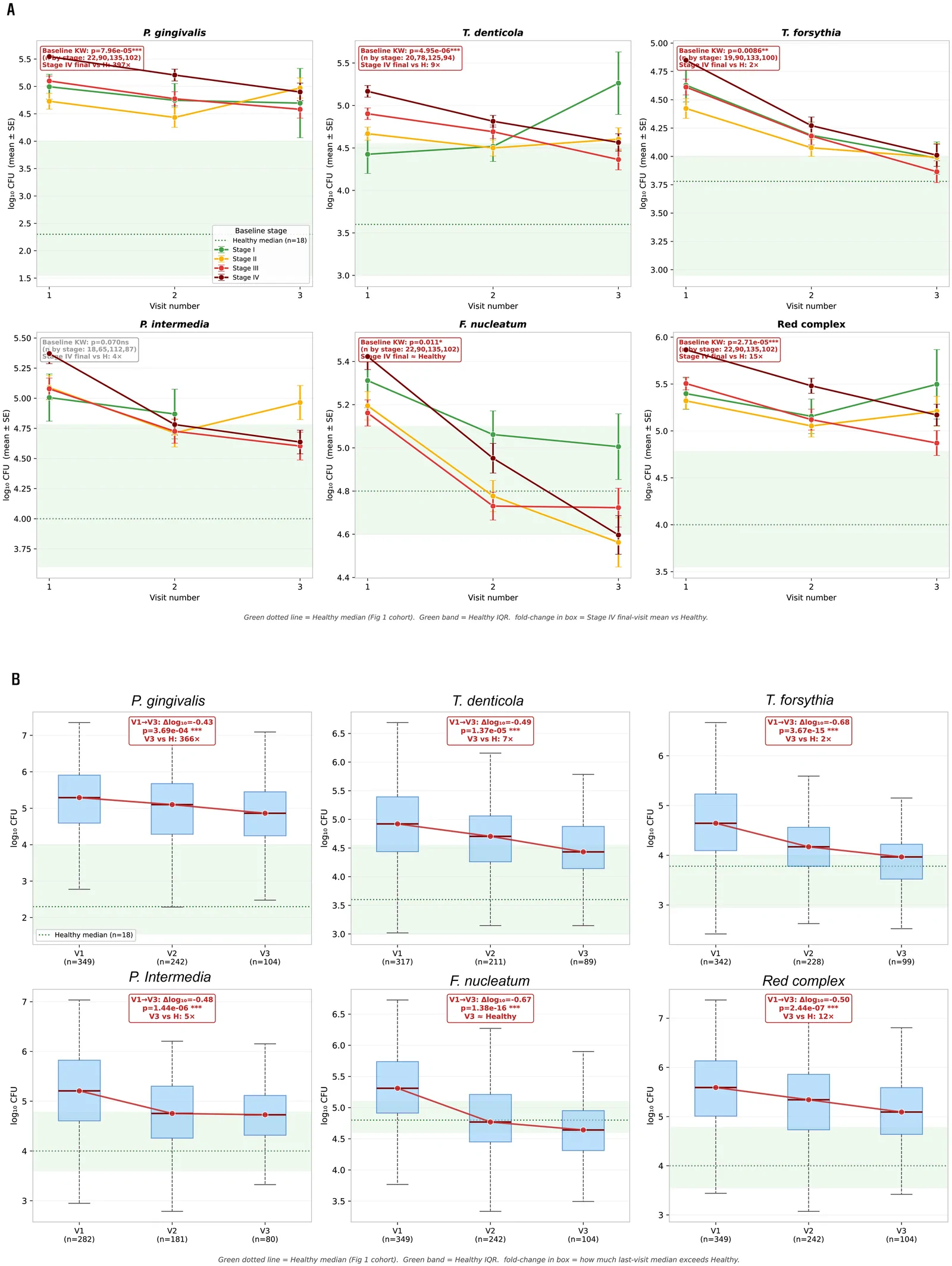
Longitudinal three-visit trajectories of pathogen burden in the real-world audit cohort. **(A)** Stage-stratified mean ± standard-error trajectories of log10 CFU across three visits (V1, V2, V3), with separate lines for each baseline AAP/EFP stage. Green dotted horizontal line indicates the Healthy reference median; green shaded band shows the Healthy interquartile range. **(B)** Pooled three-visit trajectories irrespective of baseline stage. Boxplots show V1, V2, and V3 log10 CFU distributions; red lines connect group medians. Annotation boxes report the V1→V3 median Δlog10, the paired Wilcoxon p-value, and the fold-difference between V3 and the Healthy reference median. CFU, colony-forming units; SE, standard error.

Because each patient served as their own baseline, these reductions reflect individual-level change rather than group averages. Target-specific evaluable denominators for the full paired analysis varied because paired V1–final values and below-limit-of-detection handling differed by species; denominators are therefore reported separately for each target in **Figure 4** and **Supplementary Figure 3**. Progressive reduction was sustained across three repeated visits (V1→V3; **Figure 5**), with median V1→V3 Δlog10 ranging from −0.43 (*P. gingivalis*) to −0.68 (*T. forsythia*), confirming that the downward trajectory was maintained, not a single-timepoint artifact.

### 3.8 Treatment response was species-specific and stage-dependent

The magnitude of response scaled with baseline severity (**Figure 4B**). For Stage IV at baseline, median Δlog_10_ reductions ranged from −0.58 (*T. denticola*) to −0.86 (*F. nucleatum*), all paired-Wilcoxon p < 10^−5^. The Jonckheere–Terpstra test for trend in treatment effect across baseline stage was significant for *T. denticola* (z = +2.16, p = 0.031), *T. forsythia* (p = 0.029), *P. intermedia* (p = 0.045), and *F. nucleatum* (p = 0.0066), consistent with a “higher-burden, larger-response” relationship.

Critically, the paired monitoring results showed that treatment response was not uniform across species. Red-complex species and ecologically supportive co-markers all decreased after therapy, but the magnitude of reduction and recovery toward the Healthy reference range differed by target—information unavailable from clinical or radiographic assessment alone. Because baseline risk-factor analysis identified dental implants as the only subgroup consistently associated with elevated V1 microbial burden, we next examined whether the same individual-level monitoring signal was preserved in this clinically enriched high-burden subgroup.

### 3.9 Dental implants identify a high-baseline-burden subgroup with trackable treatment response

**Figure 6:**
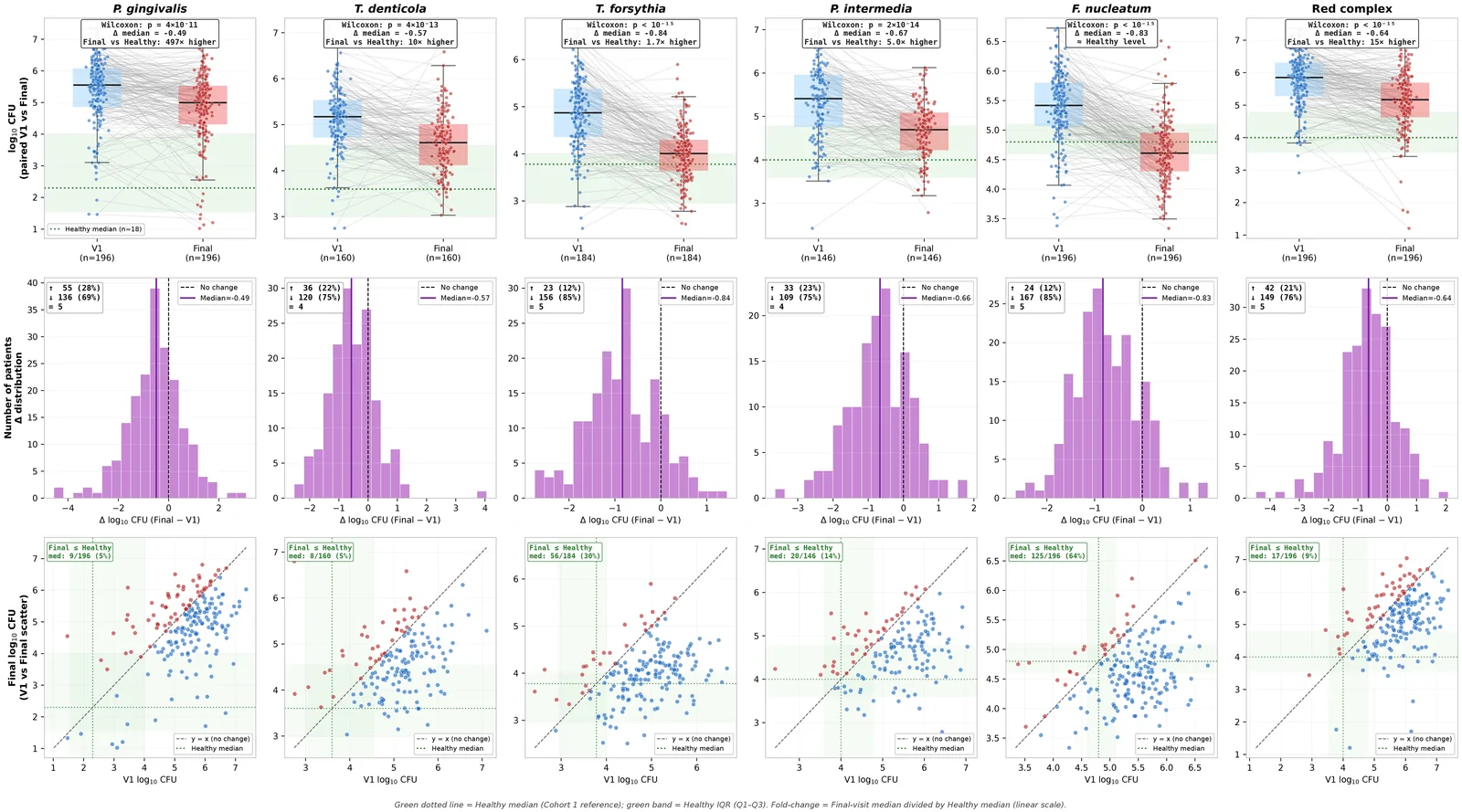
Treatment-response monitoring in the implant-bearing high-baseline-burden subgroup (V1 vs final visit). Among nine baseline patient risk factors examined in the longitudinal cohort, the presence of at least one dental implant was the only factor consistently associated with elevated baseline V1 CFU loads. This figure shows individual-level V1 versus final-visit pathogen burden among implant-bearing patients with target-specific evaluable paired measurements. Top row, paired V1 versus final-visit log10 CFU boxplots with within-subject linking lines; annotation boxes report Wilcoxon signed-rank p-values, median Δlog10 CFU, and the fold-difference between the final-visit median and the Healthy reference median from Cohort 1 on the linear CFU scale. The green dotted horizontal line indicates the Healthy median, and the green shaded band shows the Healthy interquartile range. Middle row, histograms of within-subject Δlog10 CFU; the dashed vertical line at 0 marks no change, and the solid purple line marks the subgroup median. Bottom row, paired scatter plots of final versus V1 log10 CFU; the dashed diagonal denotes y = x, and points below the diagonal indicate post-treatment reduction. CFU, colony-forming units. Target-specific evaluable denominators ranged from n = 144 to n = 196 after target-specific availability and below-limit-of-detection handling.

Among nine baseline patient risk factors examined in the longitudinal cohort (**Supplementary Figure 8**), the presence of at least one dental implant was the only factor consistently associated with elevated baseline V1 CFU loads, reaching nominal Mann–Whitney U p < 0.05 for four of five species. We therefore examined this implant-bearing subgroup as a clinically enriched high-burden subgroup for treatment-response monitoring. Within the implant-bearing subgroup, all five pathogens showed significant V1 → final-visit reductions after documented periodontal therapy, with target-specific evaluable denominators ranging from n = 144 to n = 196 across targets after below-limit-of-detection handling; all reductions were statistically significant (least-significant paired Wilcoxon p = 4.6 × 10^−10^), with 95% confidence intervals for the median Δlog_10_ reported per target in **Figure 6**.

Residual-burden patterns remained species-specific. Post-treatment *F. nucleatum* reached or fell below the Healthy reference median in 61% of evaluable implant-bearing patients (119/196), whereas *P. gingivalis* reached the Healthy reference median in only 5% (9/196) and remained approximately 500-fold above the Healthy reference at the median final visit. These findings show that the assay can track both reduction and persistence of pathogen burden in the only baseline risk-factor subgroup with elevated microbial burden. Residual burden is reported here as a quantitative observation; whether it constitutes an actionable prognostic marker was not tested, and implant-bearing patients are identified as a clinically relevant target for future peri-implant monitoring studies rather than for immediate clinical decision-making.

## 4 Discussion

### 4.1 Principal findings

In this two-cohort evaluation, a mouthwash-based multiplex qPCR assay with absolute CFU quantification of five periodontal pathogens provided two linked clinical outputs in a single non-invasive test. First, it discriminated severe Stage III–IV periodontitis with high accuracy, achieving LOOCV AUC 0.96 for *P. gingivalis* alone and 0.97 for the combined panel. Second, using the same absolute-CFU scale, it quantified within-patient species-level microbial response to non-surgical periodontal therapy in 235 patients with paired pre- and post-treatment samples. In the implant-bearing high-baseline-burden subgroup, *F. nucleatum* reached or fell below the Healthy reference median in 61% of evaluable patients, whereas *P. gingivalis* reached it in only 5% and remained approximately 500-fold above the Healthy reference at the median final visit, revealing residual keystone-pathogen burden not captured by clinical or radiographic assessment. Absolute CFU loads rose monotonically across four ordered AAP/EFP groups (red-complex composite Jonckheere–Terpstra p = 1.5 × 10^−17^), and screening performance was preserved after adjustment for age and sex and after re-inclusion of antibiotic- or treatment-exposed subjects (LOOCV AUC 0.93; optimism 0.02), indicating robustness against the two confounders most commonly invoked against small-sample biomarker studies.

### 4.2 Dual clinical utility on a single absolute-CFU scale

The principal contribution of this work is the demonstration that a single non-invasive mouthwash test can provide two linked clinical outputs on one absolute-CFU scale: high-accuracy screening for severe periodontitis and longitudinal quantification of species-level pathogen response after therapy. Three features make this monitoring clinically meaningful rather than merely statistical. First, because the analysis is paired within patient, each subject serves as their own baseline, and the reductions observed in the full longitudinal cohort reflect individual-level change rather than between-subject differences. The repeated-visit analysis further showed that the downward trajectory was sustained across V1→V3, rather than reflecting a single-timepoint artifact. In the implant-bearing high-baseline-burden subgroup, direction of change was also consistent, with 66–84% of target-evaluable patients showing decreases depending on species (**Figure 6**). Second, the response was strongly species-specific in a way that carries direct clinical meaning. In the implant-bearing high-baseline-burden subgroup, *F. nucleatum* frequently returned to the Healthy reference range, whereas *P. gingivalis* persisted above the Healthy reference in 95% of evaluable patients. This divergence aligns with the established biological asymmetry between facultative bridge organisms that respond rapidly to mechanical biofilm disruption and obligate anaerobic keystone pathogens that persist in protected ecological niches (Mombelli, 2018). This persistence pattern is invisible to clinical or radiographic assessment: a patient judged clinically improved may still harbor a substantial residual keystone-pathogen burden, and the panel makes that burden visible and quantifiable. Third, the magnitude of reduction scaled with baseline severity (Jonckheere–Terpstra trend significant for four of five species), consistent with a “higher-burden, larger-response” relationship that the absolute-CFU readout can quantify on a fixed scale.

### 4.3 Distinction from existing periodontal pathogen assays

Several commercial and laboratory qPCR assays estimate the loads of selected periodontal pathogens, and the cross-sectional detection of red-complex species in disease is well established (Socransky et al., 1998). What distinguishes the present work is not the detection of these species per se, but the use of the same absolute-CFU mouthwash signal for two clinical functions: discriminating severe disease at a single visit and following species-specific pathogen response across visits. First, the assay reports absolute CFU on a standard-curve system cross-validated against shotgun metagenomic sequencing (Hwang et al., 2024), rather than relative abundance or semi-quantitative categories; this fixed scale is what permits direct pre/post comparison within a patient without the compositional normalization that constrains sequencing-based approaches. Second, quantification spans more than four orders of magnitude between health and severe disease, providing the dynamic range needed to register treatment-induced change rather than only presence/absence. Third, and most importantly, we validate the assay not only as a cross-sectional classifier but as a longitudinal monitoring tool in a real-world cohort an order of magnitude larger than prior mouthwash-qPCR reports, demonstrating that the same test tracks species-specific response over time. The contribution is therefore the use of a single quantitative assay for both severe-periodontitis screening and individual-level treatment monitoring, rather than for one-time pathogen detection alone.

### 4.4 Screening performance in context

The discriminative performance of *P. gingivalis* alone (LOOCV AUC 0.96) is among the highest reported for any single microbial biomarker in periodontitis screening (Dong et al., 2025), and its emergence as the strongest individual discriminator is consistent with the keystone-pathogen hypothesis (Curtis, 2012). The combined five-species model performed comparably to *P. gingivalis* alone under LOOCV (0.97 vs 0.96), and decision curve analysis indicated that the three-species red-complex composite captured most of the screening signal—suggesting that, for the screening role specifically, a parsimonious red-complex readout may suffice. These findings position screening as an immediate clinical use case of the panel, while longitudinal monitoring extends the same quantitative signal into repeated assessment of treatment response.

### 4.5 Ecological co-marker interpretation

After ANCOVA adjustment, the independent effect of *F. nucleatum* attenuated to non-significance (β = −0.08, p = 0.74) and *P. intermedia* retained a significant but considerably reduced effect (β = +1.26, p = 8 × 10^−4^) relative to the three red-complex species (β = +1.3 to +3.6). We interpret these findings as evidence that, within this panel, *F. nucleatum* and *P. intermedia* function as ecologically supportive co-markers—informative about the broader dysbiotic shift and about bridge-organism dynamics during treatment response—rather than as primary discriminators of disease stage. Notably, the same species that contribute little to cross-sectional staging (*F. nucleatum* in particular) are the most informative about treatment response, returning to health most readily; the two roles are complementary, and a panel intended for monitoring as well as screening benefits from retaining them. This interpretation is congruent with the polymicrobial synergy and dysbiosis model (Hajishengallis and Lamont, 2012) and with the community-level findings of our companion 16S rRNA sequencing study (Cho et al., 2026).

### 4.6 Strengths and limitations

Principal strengths include absolute CFU quantification on a standard-curve system independently validated against shotgun metagenomic sequencing; two complementary cohorts designed and analyzed independently; a longitudinal monitoring cohort an order of magnitude larger than prior mouthwash-qPCR reports; a pre-specified sensitivity analysis modeling real-world deployment; LOOCV with explicit optimism reporting; and adherence to STARD 2015, MIQE 2009, and STROBE frameworks. Several limitations qualify these findings. First, the cross-sectional cohort is a single-center pilot screening validation (n=87) with no external test set. Although LOOCV with optimism correction was used to reduce over-optimism, the high internal AUCs (0.96–0.97) may still be optimistic given the modest sample size, single-center recruitment, and exclusion of antibiotic- and treatment-exposed subjects from the primary analysis; the pre-specified sensitivity analysis that re-introduced exposed subjects partially addresses the last point but does not substitute for independent external validation. Multi-center external validation on an independent cohort is required before chair-side or population-level deployment, and the reported performance should be regarded as an internal upper bound pending such validation. Because age differed significantly across severity groups (**Table 1**) and is itself a recognized modifier of the oral microbiota, all between-group screening comparisons were adjusted for age (and sex) by ANCOVA; the three red-complex species and the red complex composite retained large, highly significant group effects after this adjustment (Section 3.5; **Supplementary Table 1**; **Supplementary Figures 1**, **S2**), indicating that the discrimination is not an artifact of the age imbalance between healthy and severe groups. Second, the longitudinal cohort is retrospective; treatment exposure was not randomized and the pre/post interval was variable (median 395 days, IQR 248–546), reflecting real-world recall patterns rather than the 3–4-month maintenance interval recommended for Stage III–IV disease; this variability limits causal inference, and the monitoring findings should be read as quantification of real-world change rather than as evidence of a controlled treatment effect. Third, eCAL-based staging was derived from panoramic AI rather than direct clinical CAL. Panoramic radiographs are known to underestimate interproximal bone loss, and eCAL is not a validated surrogate for clinical attachment level; we therefore do not claim equivalence between eCAL and direct CAL measurement. eCAL was used only as an operational severity classifier in Cohort 2, where direct periodontal measurements were unavailable, and its performance was benchmarked against clinician staging in Cohort 1 (quadratic-weighted κ = 0.84; 94.6% sensitivity and 96.2% specificity for the severe/non-severe boundary), from which we estimate ~4–5% misclassification across that boundary. This measurement-error attenuation in the reference standard would, if anything, bias screening performance toward the null. It should also be noted that under the 2018 AAP/EFP framework, tooth loss due to periodontitis is itself a component of severity-based staging (Stage III, ≤4 teeth lost; Stage IV, ≥5 teeth lost) rather than an independent confounder of the microbial measurement. Consistent with this, remaining teeth decreased across stages in Cohort 1 (**Table 1**), and because the whole-mouth mouthwash sample integrates organisms from all remaining dentate and peri-implant surfaces, the reduced number of measurable sites in the most advanced stage contributes to the marginally lower CFU medians observed in Stage IV relative to Stage III for several targets (**Table 1**, note); this reflects fewer colonized sites at equal or greater disease severity rather than lower microbial burden per site, and does not alter the monotonic CFU increase across the ordinal severity gradient tested by the Jonckheere–Terpstra trend. Fourth, the panel quantifies five pre-specified species and does not capture broader community-level dysbiosis. Fifth, the monitoring analyses quantify microbial change but were not linked to longitudinal clinical endpoints (probing depth, attachment level, or inflammation resolution) at the paired-visit level, and we did not test whether microbial trajectories predict clinical recurrence; the clinical significance of a given residual burden therefore remains to be established, and the species-level trajectories reported here are observations rather than validated prognostic or decision-guiding markers. Although the underlying CFU-based standard-curve assay was previously validated for linearity and quantitative accuracy (Section 2.4; Hwang et al., 2024), its inter-run and lot-to-lot analytical precision were not separately characterized in the present cohorts and should be established in analytical-validation work supporting routine deployment. We also did not assess day-to-day or within-day biological variability of the mouthwash signal in the same individual; because sampling was standardized (fixed rinse volume, duration, and post-brushing timing) on a fixed absolute-CFU scale, we expect such variability to be modest relative to the several-order-of-magnitude differences between health and disease and the treatment-associated changes reported here, but formal test–retest reproducibility of repeated sampling was not evaluated and warrants dedicated study. The implant-bearing subgroup analysis was exploratory: implant status was analyzed as a baseline risk factor, not as a diagnosis of peri-implantitis, and implant-specific probing depths, peri-implant bone-loss endpoints, or peri-implantitis treatment outcomes were not available.

### 4.7 Clinical and translational implications

These findings support positioning the five-pathogen mouthwash multiplex qPCR panel as a dual screening and monitoring adjunct that complements, not replaces, clinical and radiographic diagnosis under the 2018 AAP/EFP framework: one-time severe-periodontitis screening to identify individuals warranting full periodontal assessment, and repeated absolute-CFU monitoring of treatment response and residual burden over time. The implant-bearing subgroup was the only baseline risk-factor subgroup with elevated microbial burden and nevertheless showed trackable V1 → final-visit reductions across all five pathogens (**Figure 6**). This finding supports implant-bearing patients as a clinically relevant high-burden group for future peri-implant microbial monitoring, although dedicated validation with implant-specific clinical endpoints will be required. Realizing these applications will require multi-center external validation, head-to-head comparison with established subgingival sampling, cost-effectiveness evaluation, and prospective trials assessing whether incorporating quantitative microbial monitoring into post-treatment maintenance care improves long-term clinical outcomes.

## 5 Conclusion

A single non-invasive mouthwash multiplex qPCR assay providing absolute CFU quantification of five periodontal pathogens delivered two linked clinical outputs: high-accuracy severe-periodontitis screening and quantitative species-specific monitoring of microbial response to therapy. In implant-bearing patients—the only baseline risk-factor subgroup with elevated microbial burden—the assay also tracked significant treatment-associated reductions, supporting future validation for peri-implant microbial monitoring. The panel is positioned as a non-invasive adjunct to the 2018 AAP/EFP framework; multi-center external validation is the immediate next step.

## Data Availability

The datasets analyzed for this study are not publicly available because they were derived from de-identified electronic medical records obtained under institutional review board approval at Apple Tree Dental Hospital and contain potentially re-identifiable clinical and microbiological information; sharing is restricted by the applicable data-protection regulations and the terms of the institutional review board approval. Requests to access anonymized summary data may be directed to the first author and will be considered on a case-by-case basis, subject to institutional approval. Requests to access these datasets should be directed to djawpgus@appleden.com.
