# Supplementary material for: Absolute quantification by mouthwash testing screens severe periodontitis and tracks species-specific microbial response to periodontal therapy
Source: Front Cell Infect Microbiol. 2026 Sep 3;16:1917870. doi: 10.3389/fcimb.2026.1917870 (PMC13581565; doi:10.3389/fcimb.2026.1917870)
Supplement: Supplementary file 1 [file DataSheet1.zip › Supplementary figures - 복사본/Supplementary_TableS1&Supplementary_Figures_S1-S9_legends.docx]

**Supplementary Table S1. ANCOVA-adjusted differences in log₁₀ CFU between healthy controls and Stage III/IV periodontitis after adjustment for age and sex (main analysis cohort, n=87).**

Tabulated ANCOVA results for the five species and the red complex composite. Columns: per-species sample size (Healthy / Periodontitis); unadjusted Mann–Whitney U p-value; ANCOVA p-values after age adjustment alone and after combined age + sex adjustment; Benjamini–Hochberg false-discovery-rate q-value (across five species, age + sex adjusted); group effect β (estimated difference in log₁₀ CFU, Stage III/IV − Healthy) with 95 % bootstrap confidence interval (2,000 iterations); p-value for the sex covariate; and model R². Red complex composite = log₁₀-sum of P. gingivalis + T. denticola + T. forsythia. Significance levels: *** p < 0.001; ** p < 0.01; * p < 0.05; ns, not significant. ANCOVA, analysis of covariance; CFU, colony-forming units; FDR, false discovery rate.

**Supplementary Figure S1. Forest plot of ANCOVA group effects (Stage III/IV vs Healthy) adjusted for age and sex (n=87).**

Point estimates and 95 % bootstrap confidence intervals (2,000 iterations) for the group-effect coefficient β from the ANCOVA model log₁₀(CFU) ~ Group + Age + Sex. Blue markers and intervals indicate species reaching significance (p < 0.05) after age + sex adjustment; grey indicates non-significance. β represents the adjusted between-group difference in log₁₀ CFU. *** p < 0.001. CFU, colony-forming units.

**Supplementary Figure S2. Effect of age and sex adjustment on Spearman partial correlations between bacterial load and disease group (n=87).**

Bar plot of Spearman ρ between log₁₀ CFU and binary group (Healthy vs Stage III/IV) under three model specifications: raw (unadjusted, red), age-adjusted (orange), and age + sex-adjusted (blue). Asterisks above blue bars indicate statistical significance after age + sex adjustment. Percentage labels above selected bars indicate the relative attenuation of ρ between the raw and adjusted models. Dotted horizontal line denotes the weak–moderate correlation threshold (ρ = 0.30). The largest attenuation is observed for F. nucleatum (–68 %), consistent with its role as an age- and ecology-dependent co-marker. CFU, colony-forming units.

**Supplementary Figure S3. Pre- versus post-treatment log₁₀ CFU in the longitudinal cohort, all-stage pooled (n up to 235 paired samples).**

Paired boxplots of pre-treatment (Pre-Tx, blue) and post-treatment (Post-Tx, red) log₁₀ CFU for the five species and the red complex composite. Connecting lines link within-subject Pre/Post observations. Annotation boxes report the median Δlog₁₀ and the paired Wilcoxon signed-rank p-value. Per-species n reflects evaluable paired samples after exclusion of BLOD pairs. Median Pre→Post interval was 395 days (IQR 248–546). BLOD, below limit of detection; CFU, colony-forming units; SRP, scaling and root planing; Tx, treatment.

**Supplementary Figure S4. Validation of the AI eCAL-based staging algorithm against clinician-assigned periodontal stage in Cohort 1 (n=109).**

Clinician-assigned stage, derived from chairside probing depth and bleeding-on-probing — measurements that are independent of panoramic radiographic imaging — served as the reference standard. eCAL-based stage was assigned from per-subject maximum estimated clinical attachment loss (cal_max) using the cutoffs ≤2 mm (Healthy/Gingivitis), 2–4 mm (Stage I–II), and >4 mm (Severe Stage III–IV). (A) Confusion matrix of eCAL versus clinician classifications; row percentages refer to the clinician-assigned category. Exact agreement 76.1%, adjacent (±1) agreement 100%, linear-weighted κ = 0.74, quadratic-weighted κ = 0.84. For severe-versus-non-severe classification, eCAL sensitivity was 94.6% (53/56) and specificity 96.2% (51/53). (B) Distribution of eCAL (per-subject maximum) by clinician-assigned stage; box plots show median and IQR with individual subjects overlaid. Dashed horizontal lines mark the applied stage cutoffs. The Jonckheere–Terpstra trend test confirmed a strong monotonic increase in eCAL across clinician stages (z = +9.56, p < 0.001).

**Supplementary Figure S5. Cutoff selection verification for the combined five-species LOOCV model under two threshold-selection rules in the main and sensitivity cohorts.**

Performance metrics (sensitivity, specificity, PPV, NPV, accuracy, F1) at the LOOCV-derived operating point under two cutoff-selection rules: the Youden index (maximizing sensitivity + specificity – 1; left column) and the balanced rule (minimizing |sensitivity – specificity|; right column), applied to the main cohort (n=87; top row) and the sensitivity analysis cohort (n=95; bottom row). Inset boxes report the threshold probability, true-positive / true-negative / false-positive / false-negative counts, the Youden index, and the |sens − spec| balance metric. The Youden-optimal threshold (top-left panel; threshold = 0.432) is the operating point reported in the main analysis. F1, F1-score; FN, false negative; FP, false positive; LOOCV, leave-one-out cross-validation; NPV, negative predictive value; PPV, positive predictive value; TN, true negative; TP, true positive.

**Supplementary Figure S6. Sensitivity analysis: ROC performance with re-inclusion of subjects with antibiotic or periodontal-treatment exposure (n=95: 32 healthy, 63 Stage III/IV).**

ROC curves for the combined five-species training and LOOCV models and for the three individual red-complex species in the expanded cohort, with AUC and 95 % bootstrap confidence intervals (2,000 iterations). The red star marks the LOOCV operating point of the combined model (sensitivity 0.92, specificity 0.81). The estimated optimism (training minus LOOCV AUC) is annotated at the lower-left. Re-introduction of 8 antibiotic- or treatment-exposed subjects preserves discriminative performance relative to the main analysis. AUC, area under the curve; CI, confidence interval; LOOCV, leave-one-out cross-validation; ROC, receiver operating characteristic.

**Supplementary Figure S7. Multivariable logistic-regression odds ratios for severe periodontitis (Stage III/IV vs Healthy) per 1 SD increase in log₁₀ CFU (n=87).**

Forest plot of odds ratios (OR) on a logarithmic x-axis with 95 % bootstrap confidence intervals (2,000 iterations) from a single multivariable logistic-regression model (L2-regularized) containing all five species as forced predictors, each standardized to unit SD. Red markers and intervals indicate species whose 95 % CI excludes 1 (significant independent contribution); grey indicates non-significant species. The three red-complex species emerge as independent significant predictors (OR 2.84–3.68), whereas F. nucleatum and P. intermedia do not retain independent significance after mutual adjustment. CFU, colony-forming units; CI, confidence interval; OR, odds ratio.

**Supplementary Figure S8. Baseline (V1) log₁₀ CFU stratified by patient-level risk factor in the longitudinal cohort (n=237).**

Boxplots of V1 log₁₀ CFU for the five species and the red complex composite (columns) across binary patient-level risk factors (rows): diabetes mellitus, hypertension, osteoporosis, cancer, autoimmune disease, current smoking, presence of dental implant(s), and prior periodontal surgery. Within each panel, per-group n is shown below the x-axis and the Mann–Whitney U p-value is annotated above; significant comparisons (p < 0.05) are highlighted in red. Presence of dental implants was associated with higher baseline burden for P. gingivalis, T. forsythia, F. nucleatum, and the red complex composite, whereas systemic comorbidities and smoking did not reach significance at this sample size. CFU, colony-forming units; DM, diabetes mellitus; HTN, hypertension.

**Supplementary Figure S9. BLOD sensitivity analysis for the paired treatment-response cohort: treatment-associated reductions are unchanged across three below-limit-of-detection handling rules.**

Median within-patient change in log₁₀ CFU (Δlog₁₀ CFU, Visit 1 → final visit) for each of the five species and the red complex composite, computed under three below-limit-of-detection (BLOD) handling rules: BLOD excluded (treated as missing; blue), BLOD imputed at one-half the species-specific LOD (orange), and BLOD floored at 1 CFU (green). For every target the three rules yield near-identical median reductions, and all estimates lie to the left of the dashed vertical line at zero (no change), indicating consistent within-patient decreases after therapy regardless of BLOD handling. Only the red complex composite shows any between-rule difference (−0.445 under the excluded rule, in which four pairs containing a below-LOD component are dropped, versus −0.452 under the imputed and floored rules, in which those pairs are retained); all single-species estimates are identical across rules because no paired value fell below the LOD. The paired cohort analysed here (n=236) differs by one boundary case from the main-text paired cohort (n=235) owing to date-ordering of the treatment filter; this does not affect any conclusion. Corresponding median values, pair counts, and paired Wilcoxon p-values are given in Supplementary Table S4. BLOD, below limit of detection; CFU, colony-forming units; LOD, limit of detection.
