# Supplementary material for: Absolute quantification by mouthwash testing screens severe periodontitis and tracks species-specific microbial response to periodontal therapy
Source: Front Cell Infect Microbiol. 2026 Sep 3;16:1917870. doi: 10.3389/fcimb.2026.1917870 (PMC13581565; doi:10.3389/fcimb.2026.1917870)
Supplement: Supplementary file 1 [file DataSheet1.zip › Supplementary figures - 복사본/Supplementary_TableS2.docx]

**Supplementary Table S2.** Discriminative performance (area under the receiver operating characteristic curve, AUC) for single-species and composite scores in the primary and sensitivity cohorts.

| **Score** | **n** | **Apparent AUC (95% CI)** | **LOOCV AUC (95% CI)** | **Optimism** |
| --- | --- | --- | --- | --- |
| **Primary cohort (n = 87)** | | | | |
| *P. gingivalis* alone | 72 | 0.98 (0.94–1.00) | 0.96 (0.91–1.00) | 0.02 |
| *T. denticola* alone | 77 | 0.92 (0.81–0.99) | 0.91 (0.80–0.98) | 0.01 |
| *T. forsythia* alone | 79 | 0.94 (0.88–0.99) | 0.94 (0.87–0.98) | 0.01 |
| *P. intermedia* alone | 59 | 0.83 (0.70–0.94) | 0.79 (0.64–0.92) | 0.04 |
| *F. nucleatum* alone | 85 | 0.77 (0.65–0.86) | 0.73 (0.62–0.84) | 0.03 |
| Red complex composite | 87 | 0.98 (0.96–1.00) | — | — |
| Orange complex composite | 87 | 0.86 (0.77–0.93) | — | — |
| 5-species total CFU sum | 87 | 0.98 (0.94–1.00) | — | — |
| Combined 5-species logistic model | 87 | 0.99 (0.96–1.00) | 0.97 (0.92–1.00) | 0.02 |
| **Sensitivity cohort (n = 95)** | | | | |
| *P. gingivalis* alone | 78 | 0.98 (0.94–1.00) | 0.97 (0.93–1.00) | 0.01 |
| *T. denticola* alone | 84 | 0.92 (0.84–0.98) | 0.91 (0.82–0.98) | 0.01 |
| *T. forsythia* alone | 85 | 0.95 (0.89–0.99) | 0.94 (0.89–0.99) | 0.01 |
| Combined 5-species logistic model | 95 | 0.95 (0.91–0.99) | 0.93 (0.87–0.97) | 0.02 |

*Note.* Discrimination of Stage III–IV vs Healthy by logistic regression with log 10 CFU as forced predictor(s) without variable selection. Apparent AUC reported with 2,000-iteration bootstrap 95% confidence interval. LOOCV, leave-one-out cross-validation. Optimism = apparent AUC − LOOCV AUC, per Steyerberg et al. (2010). The "n" column denotes evaluable n after exclusion of below-limit-of-detection observations (single-species analyses); combined and composite models used half-LOD imputation per MIQE recommendations (n = 87 primary; n = 95 sensitivity). Red complex composite = log 10 sum of P. gingivalis + T. denticola + T. forsythia CFU loads; orange complex composite = log 10 sum of P. intermedia + F. nucleatum. Sensitivity cohort re-introduces 8 subjects whose clinical profiles would have qualified for the primary cohort's anchor groups in the absence of antibiotic or periodontal-treatment exposure exclusion.
