# Supplementary material for: Absolute quantification by mouthwash testing screens severe periodontitis and tracks species-specific microbial response to periodontal therapy
Source: Front Cell Infect Microbiol. 2026 Sep 3;16:1917870. doi: 10.3389/fcimb.2026.1917870 (PMC13581565; doi:10.3389/fcimb.2026.1917870)
Supplement: Supplementary file 1 [file DataSheet1.zip › Supplementary figures - 복사본/Supplementary_TableS3.docx]

**Supplementary Table S3. Primer and TaqMan Probe Sequences Used for Multiplex qPCR**

FAM = 6-carboxyfluorescein; JOE = 6-carboxy-4′,5′-dichloro-2′,7′-dimethoxyfluorescein; TAMRA = 6-carboxytetramethylrhodamine; BHQ1/2 = Black Hole Quencher 1/2.
All reactions were performed on a QuantStudio 3 Real-Time PCR System (Thermo Fisher Scientific, USA).

| **Multiplex Set** | **Target Species** | **Target Gene** | **Primer / Probe** | **Sequence (5′→3′)** | **Label** |
| --- | --- | --- | --- | --- | --- |
| **Set 1** | ***T. denticola*** | 16S rRNA | Forward | CCGAATGTGCTCATTTACATAAAGGT | — |
|  |  |  | Reverse | GATACCCATCGTTGCCTTGGT | — |
|  |  |  | Probe | ATGGGCCCGCGTCCCATTAGC | FAM–TAMRA |
|  | ***C. rectus*** | 16S rRNA | Forward | TCTTTACATAAGTTAAGTCGGG | — |
|  |  |  | Reverse | TTACGCTCCGAAAAGTGTCA | — |
|  |  |  | Probe | AGTTTTTCGGTGTAGGATGAGGCT | JOE–BHQ1 |
| **Set 2** | ***T. forsythia*** | 16S rRNA | Forward | ATCCTGGCTCAGGATGAACG | — |
|  |  |  | Reverse | TACGCATGCCCATCCGCAA | — |
|  |  |  | Probe | ATGTAACCTGCCCGCAACAGAGGGATAAC | FAM–TAMRA |
|  | ***P. intermedia*** | 16S rRNA | Forward | AGTCGAGGGGAAACGGCAT | — |
|  |  |  | Reverse | CTTTGGTCCACGTCAGATGC | — |
|  |  |  | Probe | AGACGGCCTAATACCCGATGTTGT | TAMRA–BHQ2 |
| **Set 3** | ***P. gingivalis*** | 23S rRNA | Forward | CTGCGTATCCGACATATC | — |
|  |  |  | Reverse | GGTACTGGTTCACTATCG | — |
|  |  |  | Probe | AGACATCCTGTGTGAATTGGCG | FAM–TAMRA |
|  | ***F. nucleatum*** | 16S rRNA | Forward | ACCTTACCAGCGTTTGACAT | — |
|  |  |  | Reverse | TGTAGCCCAGCGTATAAGGG | — |
|  |  |  | Probe | CCTAAAGACAGGTGGTGCATGGCT | JOE–BHQ1 |
|  | ***S. mutans*** | gtfD | Forward | CAGCGATAAGACAGCCTATGCTAA | — |
|  |  |  | Reverse | TGGTTTACCCGTTTGACTGGTT | — |
|  |  |  | Probe | CCGATTACCGTCTTTTGAACCGCACA | TAMRA–BHQ2 |
| **Set 4** | Total bacteria | 16S rRNA (V4–V5) | Forward | CCAGCAGCCGCGGTAATACG | — |
|  |  |  | Reverse | CCGTCAATTCMTTTRAGTTT | — |
|  |  |  | Probe | TACCAGGGTATCTAATCC | FAM–TAMRA |

M = A or C (IUPAC degenerate base); R = A or G. Each reaction: 20 µL total volume; primers at 200 nM, probes at 100 nM final concentration; 2× PCR Master Mix (Enzynomics or Bioneer, South Korea); template DNA 1 µL. Thermal cycling: 95°C 10 min; 40 × (95°C 15 s / 55°C 30 s / 72°C 30 s). The OBCheck multiplex assay includes additional targets (C. rectus, S. mutans, and a total-bacteria 16S reference); only the five periodontal pathogens analysed in this study (*P. gingivalis, T. denticola, T. forsythia, P. intermedia, F. nucleatum*) are the subject of the present report, and the remaining targets were not used in any analysis reported here.
