# Supplementary material for: Absolute quantification by mouthwash testing screens severe periodontitis and tracks species-specific microbial response to periodontal therapy
Source: Front Cell Infect Microbiol. 2026 Sep 3;16:1917870. doi: 10.3389/fcimb.2026.1917870 (PMC13581565; doi:10.3389/fcimb.2026.1917870)
Supplement: Supplementary file 1 [file DataSheet1.zip › Supplementary figures - 복사본/Supplementary_TableS4.docx]

**Supplementary Table S4.** BLOD sensitivity analysis for the paired treatment-response cohort (n=236). Median within-patient Δlog₁₀ CFU (V1→final), number of pairs analysed (n), and paired Wilcoxon p-value for each target under three below-limit-of-detection handling rules. Per-target n differs because the number of evaluable V1→final paired measurements varies by species; because no single-species paired value fell below the LOD, the three BLOD rules yield identical single-species estimates, and only the red complex composite (which aggregates across species) differs between rules. LOD, limit of detection.

| Species | Excluded (missing) | Imputed (½ LOD) | Floored (1 CFU) |
| --- | --- | --- | --- |
| P. gingivalis | −0.375 (n=236), p=1.4×10⁻¹⁰ | −0.375 (n=236), p=1.4×10⁻¹⁰ | −0.375 (n=236), p=1.4×10⁻¹⁰ |
| T. denticola | −0.440 (n=197), p=6.3×10⁻¹² | −0.440 (n=197), p=6.3×10⁻¹² | −0.440 (n=197), p=6.3×10⁻¹² |
| T. forsythia | −0.725 (n=224), p=2.0×10⁻²³ | −0.725 (n=224), p=2.0×10⁻²³ | −0.725 (n=224), p=2.0×10⁻²³ |
| P. intermedia | −0.533 (n=169), p=8.9×10⁻¹⁴ | −0.533 (n=169), p=8.9×10⁻¹⁴ | −0.533 (n=169), p=8.9×10⁻¹⁴ |
| F. nucleatum | −0.660 (n=236), p=9.0×10⁻²⁷ | −0.660 (n=236), p=9.0×10⁻²⁷ | −0.660 (n=236), p=9.0×10⁻²⁷ |
| Red complex | −0.445 (n=232), p=3.1×10⁻¹⁵ | −0.452 (n=236), p=4.5×10⁻¹⁶ | −0.452 (n=236), p=4.2×10⁻¹⁶ |

n=236; one boundary case differs from the main paired cohort (n=235) owing to date-ordering of the treatment filter; this does not affect any conclusion.
